# Intragenic duplication of *EHMT1* gene results in Kleefstra syndrome

**DOI:** 10.1186/s13039-014-0074-7

**Published:** 2014-10-23

**Authors:** Eva Maria Christina Schwaibold, Mateja Smogavec, Elke Hobbiebrunken, Lorenz Winter, Barbara Zoll, Peter Burfeind, Knut Brockmann, Silke Pauli

**Affiliations:** Institute of Human Genetics, Georg August University, Heinrich-Düker-Weg 12, 37073 Göttingen, Germany; Department of Pediatrics and Pediatric Neurology, Georg August University, Robert-Koch-Str. 40, 37075 Göttingen, Germany

**Keywords:** Array CGH, *EHMT1*, Haploinsufficiency, Intragenic duplication, Kleefstra syndrome, KS

## Abstract

**Background:**

Kleefstra syndrome is characterized by intellectual disability, muscular hypotonia in childhood and typical facial features. It results from either a microdeletion of or a deleterious sequence variant in the gene *euchromatic histone-lysine N-methyltransferase 1* (*EHMT1*) on chromosome 9q34.

**Results:**

We report on a 3-year-old girl with characteristic symptoms of Kleefstra syndrome. Array comparative genomic hybridization analysis revealed a 145 kilobases duplication spanning exons 2 to 10 of *EHMT1*. Sequence analysis characterized it as an intragenic tandem duplication leading to a frame shift with a premature stop codon in *EHMT1*.

**Conclusions:**

This is the first description of an intragenic duplication of *EHMT1* resulting in Kleefstra syndrome.

## Background

Kleefstra syndrome (KS; OMIM #610253) is a clinically well described genetic disorder characterized by the phenotypical core features of psychomotoric retardation/intellectual disability (ID), muscular hypotonia and characteristic facial dysmorphisms. The underlying cause of KS is - in approximately 75% - a microdeletion in the chromosomal region 9q34 leading to either a partial or an entire *EHMT1* loss [1-4]. The other causes of KS are heterozygous intragenic loss-of-function mutations in the *EHMT1* gene (~25%; OMIM *607001) [1-4]. There seems to be no clear genotype-phenotype correlation regarding patients with microdeletions in 9q34 and intragenic mutations in *EHMT1* [3,4]. Duplications of the entire or partial *EHMT1* gene have been reported [5,6] but none of these duplications was strictly intragenic and none of them led to a KS phenotype.

*EHMT1* encodes for a methyltransferase specific for lysine-9 of histone H3 and is a component of the transcription factor E2F6, which can repress gene transcription [7]. E2F6-methylation by EHMT1 is probably important for transcriptional inactivation via chromatin remodeling [7].

Here, we describe for the first time a patient with the typical symptoms of KS carrying a 145 kilobases (kb) intragenic duplication in the *EHMT1* gene*.* The submicroscopic duplication in *EHMT1* was detected by array comparative genomic hybridization (aCGH). Transcript analysis revealed a tandem duplication leading to a frame shift and a premature stop codon, suggesting haploinsufficiency as the underlying cause of KS [1,3,8].

## Case presentation

### Case report

The patient is the third child of healthy non-consanguinous parents. She has two healthy older brothers. Her mother had an intellectually disabled half-brother. Her father’s paternal uncle died directly after birth for unknown reasons. Prenatal ultrasound demonstrated a fetal constitutional growth delay, a polyhydramnion and a single umbilical artery. The girl was born spontaneously at 39 weeks gestation. Her birth length, her birth weight and her head circumference were between the 3^rd^ and the 10^th^ centile. Apgar scores were 9/9/9.

Developmental delay was first noted at 3 months of age. The girl showed marked muscular hypotonia. At 2 years of age she could sit without support but was still not able to crawl or walk at the age of 3 years.

She tended to be very quiet and did not react to sounds. A hearing test was anamnestically normal. The girl began to vocalize and starts teeth grinding when she was one year old but she could not speak at the age of 3 years.

She displayed autistic features with stereotypic movements and the vocalization of clicking voices with her tongue. At 3 years of age she attended a special nursery.

Cranial MRIs at the age of 2 and 3 years, respectively, revealed unspecific bilateral T2-hyperintense white matter changes in the occipital region. An electroencephalogram at the age of 3 years was normal.

Echocardiography demonstrated a haemodynamically irrelevant patent foramen ovale and a mild peripheral pulmonary stenosis. Myocardial function was normal.

**Figure 1:**
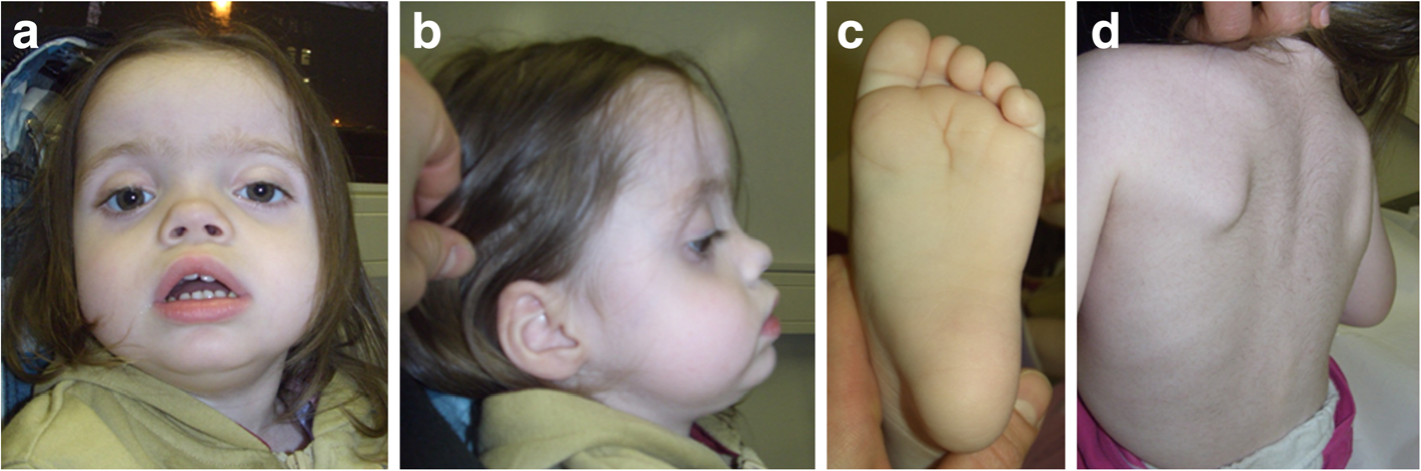
**Representative photographs of the patient at 2 11/12 years of age. (a-b)** The main facial features of the girl were: brachycephaly, prominent forehead, hypertelorism with mildly downslanting palpebral fissures, intermittent exotrophy, synophris, small nose with anteverted nostrils and deep-set nasal root, mild prognathism, deep-set posterior rotated ears, full cheeks and prominent philtrum. Note the mostly opened mouth with cupid bowed upper lip and full lower lip. **(c)** The frontal part of her plantar feet was deeply creased. **(d)** Her back was hairy.

At 2 11/12 years of age the patient displayed the following facial features (Figure 1a-b): square, brachycephalic face with a prominent forehead and frontal bossing, slight midface hypoplasia, hypertelorism with mildly downslanting palpebral fissures, synophris, small nose with anteverted nostrils and deep-set nasal root, mild prognathism, deep-set posterior rotated ears, full cheeks and prominent philtrum. The girl held her mouth mostly opened with a cupid bowed upper lip, full lower lip and a slightly protruding tongue. An ophthalmologic examination confirmed an intermittent exotrophy. The patient’s soles of the feet were deeply creased in their frontal part (Figure 1c). Her back was hairy (Figure 1d).

## Results

**Figure 2:**
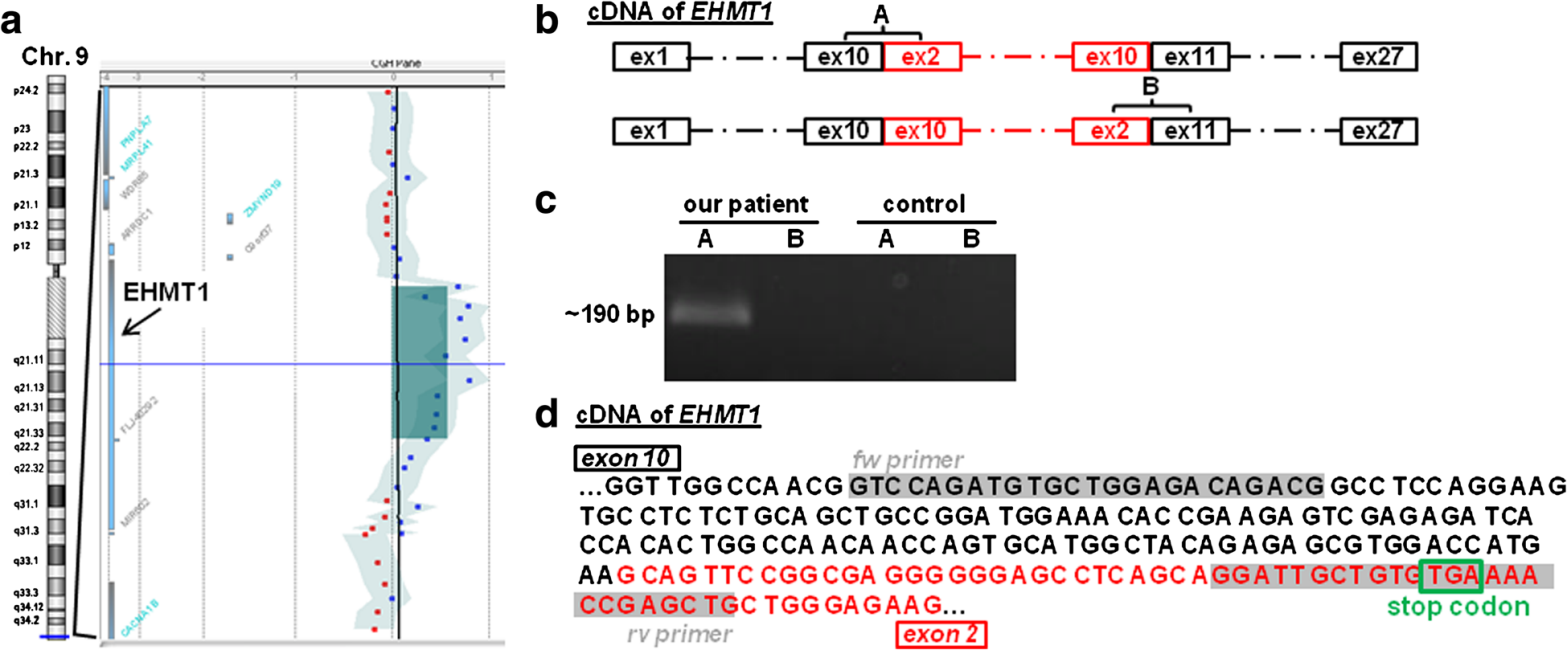
**Microduplication within** ***EHMT1*** **gene results in KS. (a)** aCGH identified a 145 kb duplication (greenish shaded; enlarged on the right side of Figure 2a) within the *EHMT1* gene (arrow) on chromosome 9q34.4. Log 2 ratio data for two dye-swap plots (patient/control) are presented according to their positions in the human genome. The light blue shaded region with blue and red dots indicates the moving average. Chr.9: chromosome 9. **(b)** Schematic overview of the two possible cDNA transcripts of *EHMT1* gene in our patient that seemed most likely. Black: normal *EHMT1* gene transcript. Red: duplicated region of *EHMT1* in our patient. ex: exon. A: indicates a possible PCR product (exon 10 of the duplicated region adjacent to exon 2 of the normal *EHMT1* transcript). B: indicates a possible PCR product (exon 2 of the duplicated region adjacent to exon 11 of normal coding *EHTM1* transcript). **(c)** Agarose gel electrophoresis of possible PCR products A and B (see Figure 2b) in our patient (first and second lane) and a control person (third and forth lane). A PCR product was only seen for A in our patient. It showed the expected size of ~190 bp of PCR product A according to the 1 kb Plus DNA Ladder (Invitrogen, Carlsbad, CA). **(d)** Sequence analysis of PCR product A (Figure 2c) and comparison with the normal coding transcript of *EHTM1* gene revealed a frameshift in the coding sequence leading to a premature stop codon (green box) in *EHMT1* in our patient. Exon 10 adjacent to exon 2 of the forward strand of *EHMT1* coding sequence in our patient is shown. Grey shaded boxes: localization of the constructed forward (fw) and reverse (rv) primers, respectively. Black: coding sequence of exon 10 of *EHMT1*. Red: coding sequence of exon 2 of *EHMT1.*

Array CGH analysis in our patient revealed a subterminal duplication on chromosome 9q34.4. The size was approximately 145 kb, spanning positions 140.535.164 to 140.657.526 (arr 9q34.3(140,527,261x2,140,535,164-140,657,526x3,140,672,499x2); GRCh37/hg19; ISCN 2013; Figure 2a). The duplication was verified by qPCR (data not shown). The parents’ array CGH analyses as well as standard karyotyping were normal (data not shown) confirming the *de novo* origin of the duplication.

The only gene in the duplicated region was *EHMT1* (Figure 2a). According to the array CGH results the centromerically located breakpoint of the duplication was within intron 1 of *EHMT1*, the terminally located breakpoint was between intron 10 and exon 13 of *EHMT1*. As microdeletions as well as sequence variants but not microduplications of or within *EHMT1* are known to cause KS and our patient’s symptoms were typical for KS the possible pathogenic background was examined.

First, the terminal breakpoint of the duplication was narrowed down to intron 10 of *EHMT1* by qPCR using exon-specific primers for the chromosomal region between positions 140.535.164 to 140.657.526. It was demonstrated that the duplication spanned exon 2 to exon 10 of *EHTM1* (data not shown). There were several possibilities regarding the localization and the orientation of the duplication but one of the two RNA transcripts shown in Figure 2b seemed most likely. Therefore, PCRs with the patient’s cDNA and primer sequences specific for the possible PCR products A (exon 10 adjacent to exon 2 of the coding *EHTM1* gene) and B (exon 2 adjacent to exon 11 of the coding *EHTM1* gene), respectively, were performed. Primers for the PCR product of exon 10 adjacent to exon 11 and the PCR product spanning exons 2 to 10, respectively, were used as positive controls for the primer function (data not shown). A PCR product was only obtained for A (Figure 2c, first lane) and for the positive controls (data not shown), not for B (Figure 2c, second lane) and not with the DNA sample of a control person (Figure 2c, third and fourth lane). By obtaining this PCR product transcript degradation that would be suggestive for nonsense mediated mRNA decay seemed to be rather unlikely. PCR product A was directly sequenced and compared with the sequences of both, exon 2 and exon 10, in wildtype *EHMT1* (ENST00000460843). The duplication within *EHMT1* resulted in a frameshift and a premature stop codon in the additionally inserted exon 2 of the *EHMT1* transcript in our patient (Figure 2d).

## Discussion

Array CGH revealed a 145 kb duplication within the *EHMT1* gene in our patient (Figure 2a). A detailed analysis of the duplicated region within *EHMT1* in our patient by qPCR and cDNA analysis revealed a direct tandem duplication of exons 2 to 10 of the *EHMT1* gene (Figure 2c).

Patients with duplications in the region of *EHMT1* have been reported [5,6] but the duplications included either the whole *EHMT1* gene, additionally the adjacent chromosomal region or one or multiple adjacent genes. Only one patient with a *EHMT1* duplication spanning exons 1 to 16 is described in the literature [5]. This patient does not display a KS phenotype but autistic features and behavioral problems [5]. The patient had a tandem duplication with both copies of *EHMT1* being probably functional. Due to the duplication in our patient a premature stop codon in the additionally inserted exon 2 is generated (Figure 2d). It is very likely that the premature termination of the protein EHMT1 will impair or reduce its function although we could not directly prove haploinsufficiency of *EHMT1*. In contrast, increased dosage of *EHMT1* - as in the other patient - might lead to neurodevelopmental impairment [5].

A possible explanation for the location of the duplication in our patient could be the existence of repetitive DNA elements in *EHMT1* in the breakpoint region of the duplication. Repetitive DNA elements in general - and *Alu* elements in particular - are prone for non-allelic homologues recombinations that can lead to disease causing chromosomal aberrations [9]. According to the RepeatMasker Web Server [10] both, intron 1 and intron 10 of *EHMT1* gene, contain high amounts of repetitive DNA elements (intron 1: 58,13% total interspersed repeats (TIRs); intron 10: 49,07% TIRs). The adjacent introns 2, 9 and 11, respectively, have lower amounts of repetitive elements. In intron 1 especially the amount of *Alu* elements that belong to the short interspersed elements was higher than in intron 2 (31,53% vs. 19.30%). The high rate of repetitive elements - especially *Alu* elements - in the breakpoint regions of the duplication in our patient provides a plausible explanation for the localization of the duplication. Likely, there will be further KS patients with deletions or duplications leading to haploinsufficiency of *EHMT1* with chromosomal breakpoints in one or both of the affected introns reported here.

Our patient displayed the typical phenotype of KS [1-4,11]; (Table 1). Only minor phenotypical differences were observed, e.g. our patient had a very hairy back and was almost underweight, both features not typically seen in KS.

**Table 1:** **Clinical findings of our patient compared with previously reported KS patients and defects in** ***EHMT1***

**Overlapping features**	**Our patient**	**Previously reported patients with KS and** ***EHMT1*** **defect (%)**
Psychomotoric retardation/ ID	+	100%
Childhood hypotonia	+	100%
Behavioural problems	+ (autistic features)	75%
Facial dysmorphisms:		
Midface hypoplasia	+	80%
Synophris	+	60%
Dysplastic/posterior rotated ears	+	50%
Short/small nose	+	45%
Brachycephaly	+	40%
Protruding tongue/macroglossia	+	40%
Hypertelorism	+	30%
Anteverted nostrils	+	25%
Tented/cupid-bowed upper lip	+	25%
Thick/everted lower lip	+	25%
Pointed chin	+	25%
**Different features**		
Overweight	-	45%
Facial dysmorphisms:		
Arched eyebrows	-	30%
Pointed chin	-	25%
Prominent forehead	+	n. r.
Neurologic defects:		
Structural CNS anomalies	-	n. r.
Seizures	-	25%
Renal anomalies	-	15%
Sensorineural hearing loss	-	15%
Deeply creased soles of the feet	+	n. r.
Hairy back	+	n. r.

+ denotes present, − denotes absent; n. r. = not reported; KS = Kleefstra syndrome; ID = intellectual disability; Table modified from [11].

## Conclusions

For the first time we could show that a duplication within the *EHMT1* gene leads to KS in a patient due to the creation of a premature stop codon in *EHMT1* that will probably impair/reduce the protein function. The gene *EHMT1* seems to be dosage sensitive with a decrease of gene expression resulting in KS and an increase of gene expression leading to a milder phenotype with an impaired neurodevelopment. The phenotype displayed by our patient is very similar compared with the previously reported KS patients and confirms the notion that there is no strong genotype-phenotype correlation in KS.

## Methods

### DNA and RNA preparation

Blood samples were collected from the patient and her parents after obtaining the parents’ signed informed consent. Total genomic DNA was prepared using standard techniques. RNA isolation was performed from a blood sample of our patient using the PAXgene™ Blood RNA kit 50v2 (PreAnalytix, Qiagen, Venlo, The Netherlands) according to the manufacturer’s instructions. cDNA was obtained using the Superscript II Kit (Invitrogen, Carlsbad, CA).

### aCGH

Genome-wide copy number scans were performed with the patient’s and her parents’ lymphocyte DNA using an Agilent SurePrint G3 Human CGH Microarray Kit 4 × 180 K and was read using an Agilent Microarray Scanner G256BA and G5761A, respectively, along with Agilent Feature Extraction Software V9.1 (Agilent Technologies, Inc., Santa Clara, CA) according to the manufacturer’s instructions. The results were analyzed using Agilent Cytogenomics 2.0 and 2.5 software, respectively. Array CGH data was confirmed by quantitative real time PCR (qPCR).

### *EHMT1* gene analysis

qPCR was used to narrow down the C-terminal breakpoint in the *EHMT1* gene by designing specific primers for exon 6 to 17 of the *EHMT1* gene (ENST00000460843). PCRs were performed to amplify the possible transcripts of the *EHMT1* gene in our patient. The obtained PCR products were directly sequenced on the ABI 3500xL Genetic Analyzer (Applied Biosystems, Foster City, CA). Their sequence was compared with the sequence of normal *EHMT1* coding transcript (ENST00000460843). All primer sequences and PCR conditions are available on request.

### Cytogenetic analysis

Metaphase chromosome spreads of blood samples of the patient’s parents were prepared from phytohemagglutinin (PHA)-stimulated peripheral blood cultures using standard protocols. 10 and 11, respectively, GTG-banded metaphases were analyzed.

## Consent

Written informed consent was obtained from the parents of the patient for publication of this Case report and any accompanying images. A copy of the written consent is available for review by the Editor-in-Chief of this journal.
